# Research Advances of Bioactive Sesquiterpenoids Isolated from Marine-Derived *Aspergillus* sp.

**DOI:** 10.3390/molecules27217376

**Published:** 2022-10-30

**Authors:** Lixiang Sun, Huannan Wang, Maocai Yan, Chunmei Sai, Zhen Zhang

**Affiliations:** 1 School of Pharmacy, Binzhou Medical University, 346 Guanhai Road, Yantai 264003, China; 2 School of Pharmacy, Jining Medical University, 669 Xueyuan Road, Rizhao 276800, China

**Keywords:** marine fungi, sesquiterpenoids, *Aspergillus*, bioactivity

## Abstract

Marine fungi *Aspergillus* sp. is an important source of natural active lead compounds with biological and chemical diversity, of which sesquiterpenoids are an extremely important class of bioactive secondary metabolites. In this paper, we review the sources, chemical structures, bioactivity, biosynthesis, and druggability evaluation of sesquiterpenoids discovered from marine fungi *Aspergillus* sp. since 2008. The *Aspergillus* species involved include mainly *Aspergillus fumigatus*, *Aspergillus versicolor*, *Aspergillus flavus*, *Aspergillus ustus*, *Aspergillus sydowii*, and so on, which originate from sponges, marine sediments, algae, mangroves, and corals. In recent years, 268 sesquiterpenoids were isolated from secondary metabolites of marine *Aspergillus* sp., 131 of which displayed bioactivities such as antitumor, antimicrobial, anti-inflammatory, and enzyme inhibitory activity. Furthermore, the main types of active sesquiterpenoids are bisabolanes, followed by drimanes, nitrobenzoyl, etc. Therefore, these novel sesquiterpenoids will provide a large number of potential lead compounds for the development of marine drugs.

## 1. Introduction

More than 70% area of the earth is covered by oceans, which is the largest known habitat for life. The marine environment is characterized by high salinity, high pressure, low oxygen, low temperature, darkness, scarce nutrients, etc. To adapt to the special environment and obtain advantages in the competition of limited resources, marine microorganisms could produce novel secondary metabolites with unique structures and potent biological activities during evolution [1,2]. Rich marine microorganisms, mainly derived from marine actinomycetes and marine fungi, are ubiquitous in the natural environment [3]. Diverse active natural products exist in endophytic fungi from the marine environment, which can be the resources for new lead compounds [4,5].

*Aspergillus* is a typical filamentous fungus, which is divided mainly into *Aspergillus fumigatus*, *Aspergillus versicolor*, *Aspergillus flavus*, *Aspergillus ustus*, *Aspergillus sydowii*, and so on [6]. Fumiquinazolines were isolated by Numata from marine *Aspergillus* sp. for the first time in 1992, which opened the door to the study of the metabolites of marine *Aspergillus* [7]. Recent studies have found that many organic compounds with unique structures, which showed a lot of physiological activities, were found in marine *Aspergillus* sp., including terpenoids, alkaloids, and polyketones [8]. Sesquiterpenoids, the most abundant among all the terpenoids skeletons, exhibit excellent biological activities, such as cytotoxicity, antibacterial, antifungal, antiviral, anti-inflammatory, and enzyme inhibitory activity, and have aroused widespread interest of many scholars [9,10]. This paper attempts to review the sources, bioactivities, biosynthesis, and other studies of sesquiterpenoids discovered from marine fungi *Aspergillus* sp. in the last 15 years.

## 2. Characteristics of Sesquiterpenoids from Marine *Aspergillus* sp.

**Figure 1:**
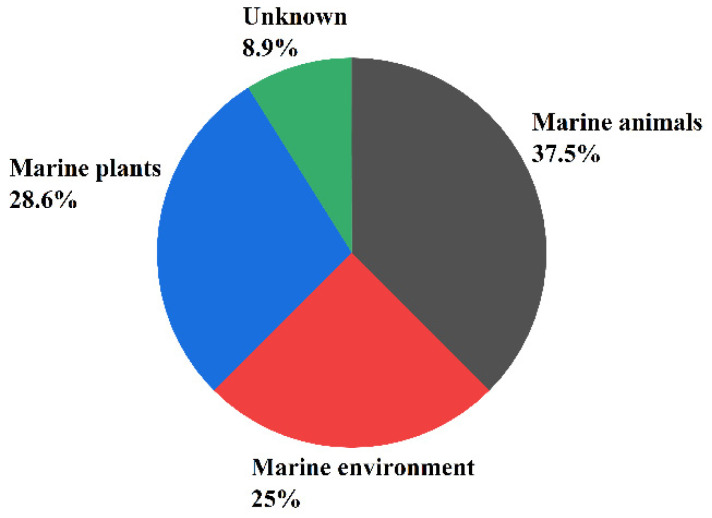
The main sources of the sesquiterpene-rich marine fungi.

Secondary metabolites of marine fungi have become one of the most active subfields of natural pharmaceutical discovery [11]. Sesquiterpenoids are an extremely important class of secondary metabolites and have been associated with a wide variety biological activities [12]. Approximately 268 sesquiterpenoids isolated from 56 strains of marine fungi are reviewed in this work. Furthermore, research has found that 37.5% of the sesquiterpenoid compounds came from marine animals (sponges, 21.4% and corals, 8.9%), 28.6% from marine plants (algae, 16.1% and mangroves, 12.5%), and the remaining compounds from the marine environment (21.4% from marine sediments and 1.8% from seawater), and 8.9% from unknown sources (see Figure 1).

**Figure 2:**
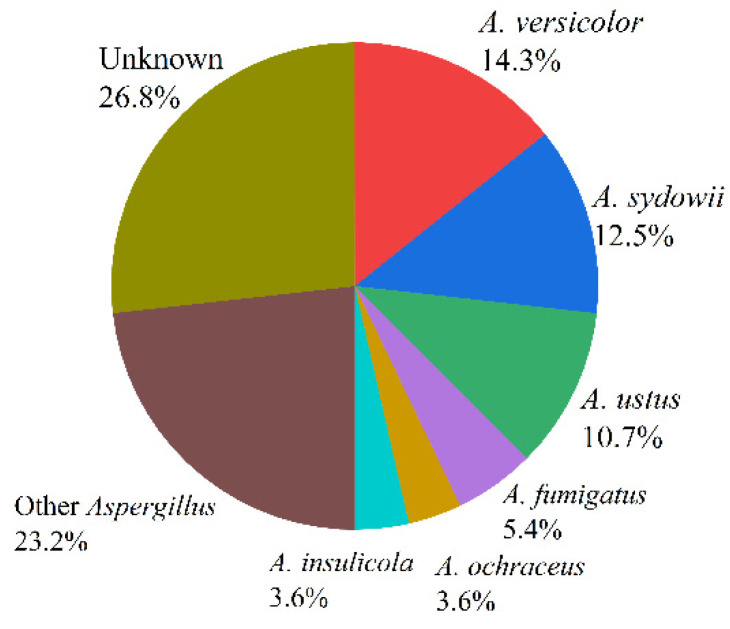
The proportions of marine fungi reviewed in this paper.

Marine fungus *Aspergillus* is a huge community that occupies a great proportion in the fungus family, which is widely distributed in marine plants, marine organisms, marine sediments, and other environments. According to incomplete statistics, there were more than 180 species of fungus *Aspergillus*, such as *Aspergillus fumigatus*, *Aspergillus flavus*, *Aspergillus terreus*, and *Aspergillus versicolor* [13]. The proportions of the 56 species (Table 1) reviewed in this paper are as follows: *Aspergillus versicolor* (14.3%), *Aspergillus sydowii* (12.5%), *Aspergillus ustus* (10.7%), *Aspergillus fumigatus* (5.4%), *Aspergillus insulicola* (3.6%), *Aspergillus ochraceus* (3.6%), *Aspergillus carneus* (3.6%), *Aspergillus terreus* (3.6%), *Aspergillus flavus* (3.6%), *Aspergillus flavipes* (3.6%), and *Aspergillus* unknown (26.8%) (see Figure 2).

**Figure 3:**
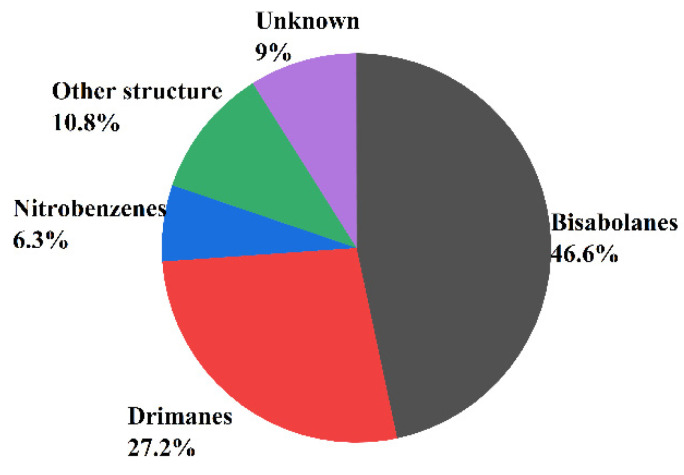
The main types of sesquiterpenoids isolated from *Aspergillus* sp.

In recent years, more and more sesquiterpenoids were found in marine fungi *Aspergillus*, which consisted of the molecular skeleton structure with three isoprene units and contains 15 carbon atoms [56]. In addition, the number and skeleton types of sesquiterpenoids are the most abundant among all the terpenoids. According to the number of carbon rings, sesquiterpenoids can be divided into acyclic sesquiterpenes, monocyclic sesquiterpenoids, bicyclic sesquiterpenoids, tricyclic sesquiterpenoids, tetracyclic sesquiterpenoids, etc., [57]. Acyclic sesquiterpenes are also known as chain sesquiterpenes but rarely reported in fungi. The monocyclic sesquiterpenes referred mainly to bisabolanes, humaranes, and cybrodins, while the bicyclic sesquiterpenes consist mainly of drimanes, lacticinanes, and eudesmanes. This paper finds that the main types of sesquiterpenoids isolated from marine fungi *Aspergillus* were bisabolanes (46.6%), drimanes (27.2%), nitrobenzenes (6.3%), and unknown structure (9%) (see Figure 3).

**Figure 4:**
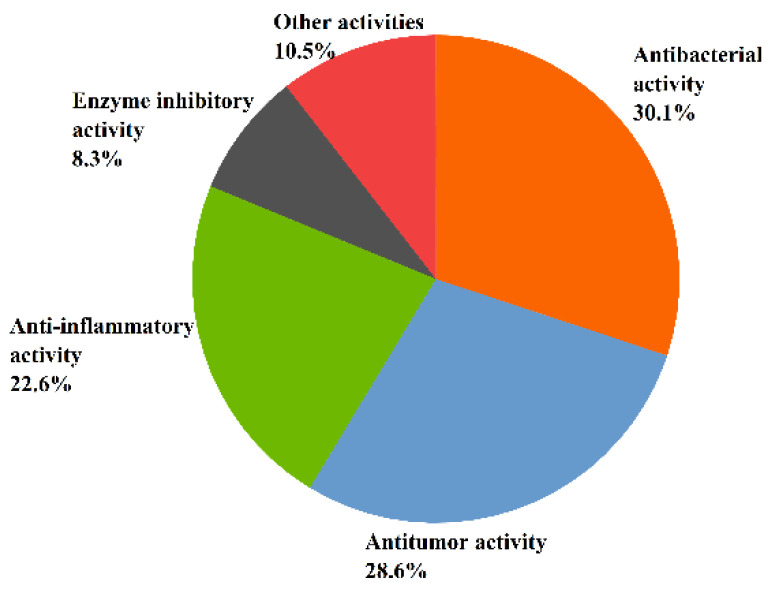
The bioactivity of sesquiterpenoids from *Aspergillus* sp.

Recent studies have indicated that the metabolic pathway of marine fungi—that results in the production of a number of secondary metabolites with various chemical structures and specific physiological activities—is very different from that of terrestrial fungi [37]. This article concludes that 131 of the 268 sesquiterpenoids isolated from marine fungi *Aspergillus* have significant biological activities. Moreover, the structure types of inactive sesquiterpenoids are mostly bisabolanes and drimanes [58,59,60,61,62]. The relatively large number of sesquiterpenoids shows a variety of biological activities such as antitumor, antibacterial, anti-inflammatory, enzyme inhibitory, antioxidant, antiviral, and other activities. Overall, 30.5% of sesquiterpenoids exhibited antibacterial activity, followed by antitumor activity (29%), anti-inflammatory activity (22.9%), enzyme inhibitory activity (8.4%), and other activities (10.7%) (see Figure 4).

## 3. Bioactivity of Sesquiterpenoids from *Aspergillus* sp.

### 3.1. Antibacterial Activity

**Figure 5:**
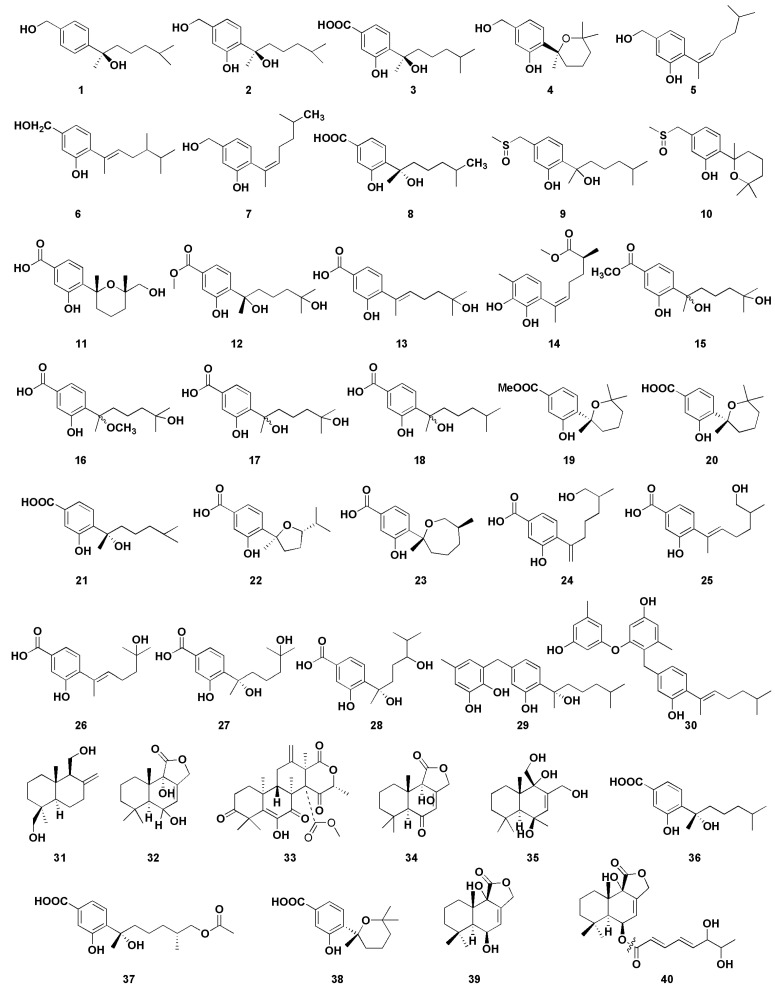
Chemical structures of antimicrobial compounds (**1**–**40**).

In recent years, inappropriate and irrational use of antibiotics provides favorable conditions for resistant microorganisms to emerge and spread, which has become a global public health problem [63]. Therefore, it is urgent to develop new antibiotics with new structures and significant biological activities. To that end, the secondary metabolites of microorganisms in the marine environment are a great source for new antibacterial agents screening and much attention has been attracted to the relevant studies. This section covers 40 bioactive sesquiterpenoids (Figure 5) with antibacterial activity described to date from marine-derived *Aspergillus* sp.

Li et al. [14] isolated four new and one known bisabolane-type sesquiterpenoid from secondary metabolites of *Aspergillus* sp. from sponge. Compounds **1**–**5** showed different antibacterial activity against six pathogenic bacteria and two marine bacteria, and compounds **2** and **4** showed selective antibacterial activity. Compound **2** had strong inhibitory effects on *Staphylococcus albus* and *Micrococcus tetragenus*, with minimum inhibiting concentrations (MIC) values of 5.00 and 1.25 µM, respectively. The MIC values of compound **4** with *S. albus* and *Bacillus subtilis* were 5.00 µM and 2.50 µM, respectively. Notably, compound **1** represents the rare example of a bisabolane-type sesquiterpenoid with a 1, 4-disubstituted benzene ring isolated from marine organisms. Compounds **2** and **3** were the enantiomers of (+)-sydonol and (+)-sydonic acid, respectively. This fact suggests that fungi isolated from different marine organisms may produce different stereochemisty compounds. Furthermore, there were three sesquiterpenoids, **6**–**8**, from the sponge-associated fungus *Aspergillus sydowii* ZSDS1-F6, which has certain antibacterial activities; among them, compound **6** and **7** displayed antibacterial activities against *Klebsiella pneumonia*, with MIC values of 21.4 and 10.7µM, respectively [15]. In addition, compound 6 showed moderate antibacterial activity against *Aeromonas hydrophila* (MIC, 4.3 µM), while compound **8** showed moderate antibacterial activity against *Enterococcus faecalis* (MIC, 18.8 µM). Chen et al. [16] isolated two phenolic bisabolane sesquiterpenoids (PBS) compounds (**9**–**10**) from *Aspergillus flavipes* 297, including a pair of new enantiomers (±)-flavilane A (**9**). However, compounds **9** and **10** represent the rare PBS-containing methylsulfinyl group and showed selective antibacterial activities against several pathogenic bacteria; their MIC values were 2–64 μg/mL. Furthermore, compound **10** exhibited mild antifungal activity against plant pathogenic fungus *Valsa mari*.

Aromatic bisabolene-type sesquiterpenoids **11**–**13** were isolated from the marine fungus *Aspergillus versicolor* SD-330 in the deep-sea sediments [17]. Compounds **11** and **12** had significant inhibitory activities against *A. hydrophilia*, *Escherichia coli*, *Edwardsiella tarda*, and *Vibrio harveyi*, with MIC values ranging from 2.0 to 8.0 μg/mL. Moreover, compound **13** had significant inhibitory activity against *E. coli* (MIC value was 1.0 μg/mL), which was better than the positive control chloramphenicol (MIC value was 2.0 μg/mL). A new aromatic bisabolene-type sesquiterpenoid (**14**) was discovered in *Aspergillus sydowii* SW9, whose absolute configuration is (*S*). Compound **14** had significant inhibitory effect on *E. coli*, and its MIC value was 2.0 µg/mL, which was similar to that of positive control chloramphenicol (MIC 2.0 µg/mL). Compound **14** also exhibited potent activity against *S. pneumonise*, with an MIC value of 4.0 µg/mL [18]. Wang et al. [19] obtained four sesquiterpenoids **15**–**18** with antibacterial activity from marine *Aspergillus versicolor* SD-330. Compounds **15** and **16** showed significant antibacterial activity against *E. coli*, *E. trada*, *V. harveyi*, and *Vibrio parahaemolyticus*, and the MIC values were less than or equal to 8.0 µg/mL. However, compound **17** exhibited significant antibacterial effect on *E. coli* with MIC value of 1.0 µg/mL, which was more potent than that of positive control chloramphenicol (MIC 2.0 µg/mL). Moreover, compound **17** showed strong inhibitory activity against *A. hydrophilia*, *E. tarda*, *Vibrio anguillarum*, and *V. harveyi*, each with MIC value of 4.0 µg/mL. Compound **17** showed a stronger antibacterial activity than compounds **15** and 16, suggesting that C-15 carboxyl group methyl ester or the methylated C-7 hydroxyl group could reduce their antibacterial activity.

Wei et al. isolated three phenolic bisabolane-type sesquiterpenoids compounds **19**–**21** from *Aspergillus* sp., which is the first report of natural metabolites from marine fungus *Aspergillus* from gorgonian *Dichotella gemmacea* [20]. All of them exhibited weak antibacterial activity against *Staphylococcus aureus,* with the diameters of inhibition zones of 11, 7, and 5 mm at 100 μg/mL, respectively. Seven phenolic bisabolane sesquiterpenoids **22**–**28** were obtained from the endophytic fungus *Aspergillus* sp. xy02 from a Thai mangrove *Xylocarpus moluccensis* [21] and displayed moderate inhibitory activities against *S. aureus*, with IC_50_ values ranging from 31.5 to 41.9 μM. Two new phenolic bisabolane sequiterpenes, asperchondols A (**29**) and asperchondols B (**30**), were obtained from the sponge-derived fungus *Aspergillus* sp. and showed antibacterial activity against *S. aureus*, with the MICs of 50 and 25 μM, respectively [22]. Furthermore, structure–activity relationship found that the coexistence of phenolic bisabolane sesquiterpene and diphenyl ether moieties seems to be very important since the hybrid **30** was more active than phenolic bisabolane sesquiterpenoid **29** and phenyl esters.

A series of phenolic bisabolane-type sesquiterpenoids have been discovered from different marine invertebrates such as sponges [64] and gorgonians [65] in the last century. In addition, such compounds were also found in bacterium CNH-741 and fungus CNC-979 isolated from marine sediments [66]. These results indicate that the real producers of these compounds from marine invertebrates, sponges, and corals may be constituents of microorganisms. Albican-11,14-diol (**31**) is a sesquiterpene compound isolated from the cultures of the endophytic fungus *Aspergillus versicolor*, which is isolated from marine green alga Codium fragile [23]. The diameters of inhibition zones of compound **31** against *E. coli* and *S. aureus* were 7 and 10.3 mm, respectively, at the concentration of 30 μg/disk. Fang et al. isolated a drimane-type sesquiterpenoid (**32**) and three unknown-type sesquiterpenoids (**33**–**35**) from the algicolos fungus *Aspergillus* sp. RR-YLW-12, which exhibited little inhibitory activity against four marine-derived pathogenic bacteria, *V. anguillarum*, *V. harveyi*, *V. parahaemolytics*, and *Vibrio splendidus* [24]. Zheng et al. isolated and purified three bisabolane sesquiterpenes **36**–**38** from the fermentation products of *Aspergillus versicolor* ZJ-2008015, which were obtained from a soft coral *Sarcophyton* sp. [25]. The results showed that compounds **36**–**38** exhibited potent antibacterial activity with MICs of 5.3, 6.4, and 5.4 μM against *S. albus* and 2.6, 6.4, and 5.4 μM against *S. aureus*, respectively. Cohen et al. [26] isolated two drimane sesquiterpenes (**39**–**40**) from the sponge-derived fungus *Aspergillus insuetus* (OY-207), which exhibited anti-fungal activity against *Neurospora crassa*, with the MICs of 140 and 242 μM, respectively.

### 3.2. Antitumor Activity

**Figure 6:**
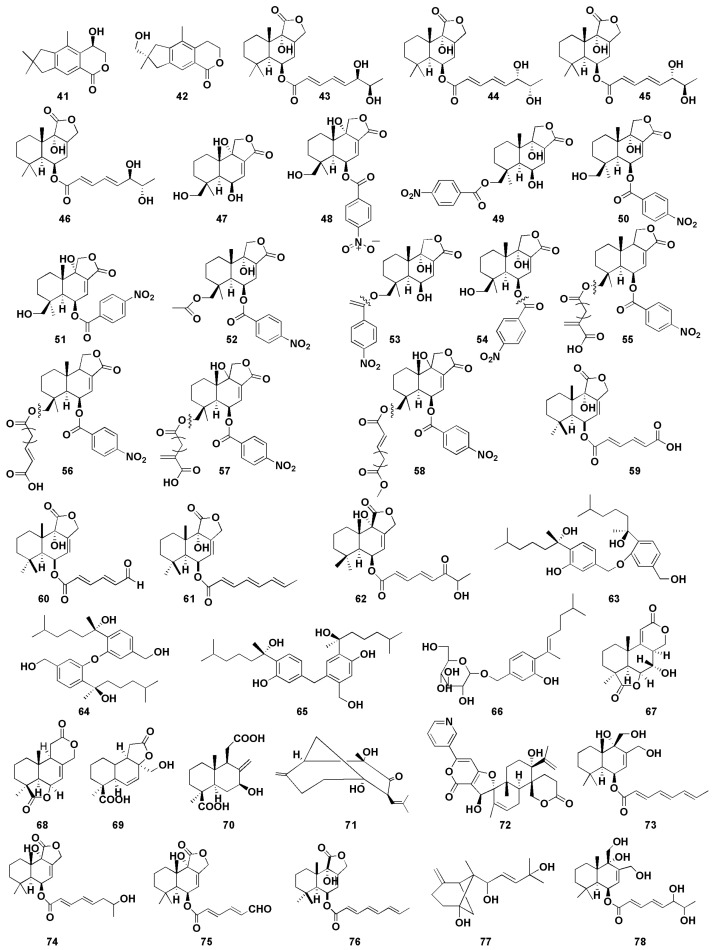
Chemical structures of antitumor compounds (**41**–**78**).

The marine environment represents a unique resource that encloses a massive chemical and biological diversity, which leads to an important source of potential antitumor drugs [67]. Among antitumor compounds, sesquiterpenes (including bisabolane, drimane, illudalane, etc.) are obtained mainly from marine fungi, including *Aspergillus* sp. [68,69]. Therefore, more and more researchers pay close attention to looking for effective antitumor drugs from marine *Aspergillus*. In recent years, there were about 38 bioactive sesquiterpenoids (Figure 6) with antitumor activity isolated from marine-derived *Aspergillus* sp.

Orfali et al. [27] first discovered two illudalane sesquiterpenes, asperorlactone (**41**) and echinolactone D (**42**), from marine sediment ascomycete *Aspergillus oryzae*, in which compound **41** has an absolute configuration of (5R). Compounds **41** and **42** showed antiproliferative activity against human lung cancer (A549), liver cancer (HepG2), and breast cancer (MCF-7) cell lines, with half maximal inhibitory concentration (IC_50_) values of asperorlactone (**41**) <100 µM. Furthermore, compounds **9** and **10** isolated from *Aspergillus flavipes* 297 exhibited promising cytotoxic effects on MKN-45 and HepG2 cells, respectively, indicating that the methylsulfinyl substituent enhanced the cytotoxicity, to a certain degree [16]. Gao et al. [28] isolated four drimane sesquiterpene esters asperienes A-D (**43**–**46**) from marine-derived fungal *Aspergillus flavus* CF13-11, which was the first successful isolation of two pairs of C-6′/C-7′ isoforms. Moreover, compounds **43**–**46** showed significant activity against four tumor cell lines (HeLa, MCF-7, MGC-803, and A549), with IC_50_ values of 1.4–8.3 µM. Notably, compounds **43** and **46** showed lower toxicity to normal GES-1 cells than did **44** and **45**, suggesting their great potential for the development of an antitumor agent. Yurchenko et al. [29] isolated two drimane sesquiterpenes (**47**–**48**) from marine-sediment-derived fungus *Aspergillus flocculosus*, which exhibited potent cytotoxic effect toward mouse neuroblastoma neuro-2A and human prostate cancer 22Rv1 cells, with the IC_50_ values were 24.1, 4.9 µM and 31.5, 3.0 µM, respectively. It is well known that human prostate cancer 22Rv1 cells are resistant to hormone therapy because of the expression of the androgen receptor splice variants AR-V7 [70]. Therefore, the results indicated that compounds **47** and **48** could be used in the treatment of human drug-resistant prostate cancer. Fang et al. [30] isolated two nitrobenzoyl sesquiterpenoids (**49**–**50**) from the marine-derived fungus *Aspergillus ochraceus* Jcma1F17, which was the first time nitrobenzoyl sesquiterpenoids obtained from this fungal were reported. Both compounds displayed significant cytotoxic effects on 10 human cancer cell lines (H1975, U937, K562, BGC-823, MOLT-4, McF-7, A549, Hela, HL60, and Huh-7), with IC_50_ values ranging from 1.95 to 6.35 µM.

Insulicolide A (Nitrobenzoyl substituted sesquiterpene, **51**) was isolated from the marine-sponge-associated endozoic fungus *Aspergillus insulicola* MD10-2 [31]. Compound **51** showed cytotoxic effects against human lung cancer cell line H-460, with an IC_50_ value of 6.9 μM. However, the cytotoxic activity of the acetylated derivatives of compound **51** decreased markedly, indicating that the double at C-7 might be involved in the cytotoxic activity. Tan et al. isolated three nitrobenzoyl sesquiterpenoids (**52**–**54**) from the marine fungus *Aspergillus ochraceus* Jcma 1F17 [32]. Compound **54** displayed potent cytotoxicities against three renal carcinoma ACHN, OS-RC-2, and 786-O cells lines (IC_50_ of 0.89–1.5 μM). The cytotoxic effects of compounds **52** and **53** on 786-O cells (IC_50_ of 2.3 and 4.3 μM, respectively) were exhibited more strongly than those of OS-RC-2 (IC_50_ 5.3 and 8.2 μM) and ACHN (IC_50_ of 4.1 and 11 μM, respectively), suggesting that the C-9 hydroxy group may contribute more to the cytotoxic activities against renal carcinoma cells. Additionally, compound **52** showed stronger inhibitory activity at low concentration levels, compared with the positive control sorafenib, a drug approved for the treatment of primary kidney cancer (advanced renal cell carcinoma). Further investigation revealed that the cell cycle was arrested at G_0_/G_1_ phase after being treated with compound **52** at 1 μM, whereas after being treated at 2 μM for 72 h, the late apoptosis of 786-O cells were induced. Four nitrobenzoyl sesquiterpenoids (**55**–**58**) were isolated from an Antarctica-sponge-derived *Aspergillus insulicola* by Sun et al. [33], in which compounds **57** and **58** showed selective inhibitory activity against human pancreatic ductal adenocarcinoma (PDAC) cell lines, whereas compounds **55** and **56** were inactive, indicating that hydroxyl groups at C-9 is essential for cytotoxicity. Furthermore, the IC_50_ values of compounds **57** and **58** against PDAC cell lines AsPC-1 and PANC-1 were 2.7, 4.6 μM and 2.3, 4.2 μM, respectively. Numerous studies have shown that most of nitrobenzoyl sesquiterpenes were obtained from the marine-derived fungus *Aspergillus ochraceus*, suggesting that *Aspergillus ochraceus* may be a good resource for the production of these compounds.

Liu et al. [34] found three drimane sesquiterpenoids (**59**–**61**) from marine sponge-derived fungus *Aspergillus ustus*, which showed cytotoxic activities against mouse lymphoma cell line L5178Y, with half maximal effective concentration (EC_50_) values between 0.6 and 5.3 μM. In addition, the EC_50_ value of compound **60** against PC12 and HeLa cells were 7.2 μM and 5.9 μM, respectively. Zhou et al. [35] isolated drimane sesquiterpenoid (**62**) from mangrove-derived fungus *Aspergillus ustus* and exhibited moderate cytotoxic effects against the mice lymphocytic leukemia P388 cell line with IC_50_ value of 8.7 μM. Sun et al. [36] isolated three bisabolane sesquiterpenoid dimers (**63**–**65**) from the sponge-derived fungus *Aspergillus* sp., and the cytotoxic activity against HePG-2 human hepatoma cell line and Caski human cervical cell line were determined in vitro. Significantly, compounds **63** and **65** with (7S) and (7′S) configuration displayed better potent cytotoxicity toward the tumor cell lines than did compound **64**. The IC_50_ values of compound **63** and **65** were 9.31, 12.40 μM and 2.91, 10.20 μM, respectively. These results suggest that the cytotoxic activity of the compound may be weakened due to the mesomeric effect since the activity of the compounds is stereoselective. β-D-glucopyranosyl aspergillusene A (**66**) from the sponge-derived fungus *Aspergillus sydowii* J05B-7F-4 exhibited mild cytotoxicity against KB (human nasopharyngeal carcinoma cells), HepG2 (human liver cancer cells), and HCT 116 (human colon cancer cells), with IC_50_ values between 50 and 70 μM [37].

Deng et al. [38] found four sesquiterpenoids containing 16 carbon atoms (**67**–**70**) from the mangrove endophytic fungus *Aspergillus terreus* GX3-3B, of which compound **67** showed inhibitory activity against human breast cancer cells (MCF-7) and human promyelocytic leukemia cells (HL-60), with the IC_50_ values were 4.49 and 3.43 μM, respectively. In addition, compound **68** exhibited promising inhibitory effect on MCF-7 cells, with an IC_50_ value of 2.79 μM, whereas compound **70** showed potent inhibitory effect on HL-60 cells, with an IC_50_ value of 0.6 μM. The structure–activity relationship indicated that the presence of C or D lactone ring may be helpful for the inhibitory against the human breast cancer cell line MCF-7. Compounds **67** and **70** showed stronger activities than did compounds 68 and 69, indicating that hydroxyl group at the C-7 position could improve the cytotoxicity toward HL-60 cell.

Aspergiketone (**71**) is the first sesquiterpenoid derivative isolated from *Aspergillus fumigatus*, which exhibited obvious cytotoxicity against HL-60 and A-549 cells, with IC_50_ values of 12.4 and 22.1 μM, respectively [39]. Oxalicine B (**72**), a unique pyridino-α-pyrone sesquiterpenoid, was obtained from the sea-urchin-derived fungus *Aspergillus fumigatus* and exhibits moderate cytotoxicity to murine P388 leukemia cells, with IC_50_ of 55.9 µM [40]. Three drimane sesquiterpenes (**73**–**75**) were isolated from marine *Aspergillus ustus* 094102 [41], of which compounds **74** and **75** showed moderate cytotoxicity against A549 and HL-60 cells, with IC_50_ values of 10.5 and 9.0 µM, respectively. Moreover, compound **73** exhibited weak cytotoxic effect to A549 and HL-60 cells, with IC_50_ values of 20.6 and 30.0 µM, respectively. Proksch et al. found a drimane sesquiterpene (**76**) from marine-sponge-derived fungus *Aspergillus ustus*, which exhibited selective inhibition on lymphoma cell line L5178Y cells (median effective dose (ED_50_), 1.9 μM) [42]. Wang et al. found a β-bergamotane sesquiterpenoids (**77**) from marine-sediment-derived fungus *Aspergillus fumigatus* YK-7, which exhibited weak inhibitory activities against U937 cells, with an IC_50_ value of 84.9 µM [43]. Asperflavinoid A (**78**), a drimane-type sesquiterpenoids, was isolated from *Aspergillus flavipes* 297 and exerted toxic effect on HepG2 and MKN-45 cells, with the IC_50_ values of 38.5 and 26.8 µM, respectively [44].

### 3.3. Anti-Inflammatory Activity

**Figure 7:**
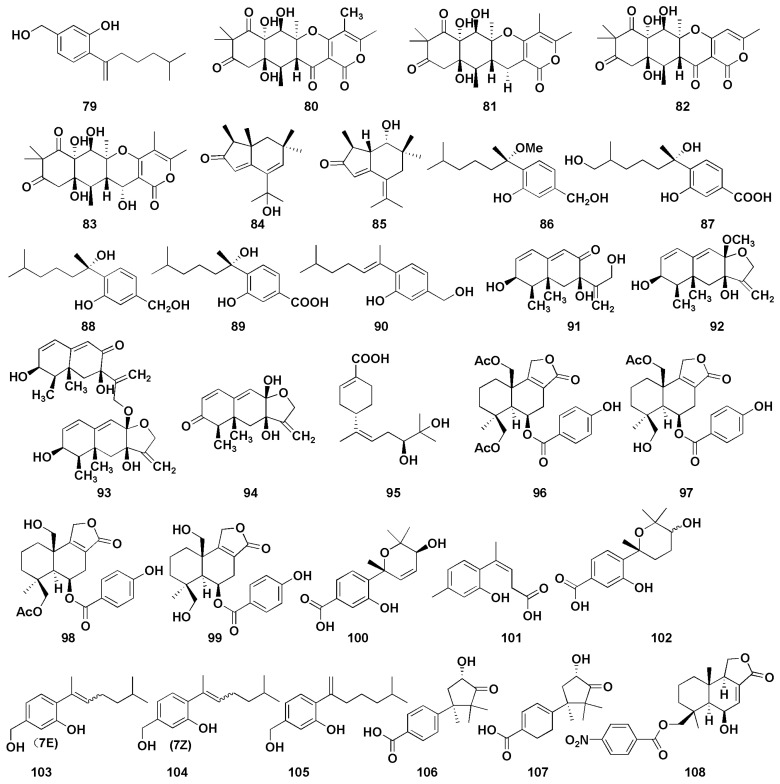
Chemical structures of anti-inflammatory compounds (**79**–**108**).

Inflammation is a comprehensive array of physiological response to a foreign organism, which has been considered as a major factor for the progression of various chronic diseases/disorders [71]. Therefore, development of effective and economical anti-inflammatory drugs (NSAIDs) is an area of importance in drug discovery while natural anti-inflammatory supplements are becoming more popular and have been the focus of many scientific investigations. This section covers 30 sesquiterpenoids (Figure 7) with anti-inflammatory activity which isolated from marine-derived *Aspergillus* sp.

Cui et al. [45] isolated a sesquiterpene derivative (**79**) from the mangrove endophytic fungus *Aspergillus versicolor* SYSU-SKS025, which was found to inhibit nitric oxide (NO) production RAW 264.7 macrophages, with an IC_50_ value of 12.5 µM (positive control, indomethacin, IC_50_ = 37.5 µM). Wang et al. [46] found four triketide-sesquiterpenoids A−D (**80**–**83**) from the marine-algal-associated fungus *Aspergillus* sp. ZL0-1B14, which exhibited anti-inflammatory activity in LPS-stimulated RAW264.7 macrophages. In addition, compound **83** inhibited the production of IL-6 with an inhibition rate of 69% at 40 μM. Wu et al. [47] firstly discovered two brasilane sesquiterpenoids (**84**–**85**) with α and β unsaturated ketones from marine-derived fungus *Aspergillus terreus*, both of which showed moderate inhibitory effects; the inhibitory rates of nitric oxide were 47.7% and 37.3%, respectively, at 40 μM. Chung et al. [48] isolated five sesquiterpenoids (**86**–**90**) with anti-inflammatory activity from *Aspergillus sydowii* in marine sediments. Among them, compounds **88** and **90** displayed selective inhibition against fMLP/CB-induced superoxide anion generation by human neutrophils, with IC_50_ values of 5.23 and 6.11 µM, respectively. At the same time, they also exhibited the most potent inhibitory activity against the release of elastase induced by fMLP/CB, with the IC_50_ values of 16.39 and 8.80 µM, respectively. Interestingly, the anti-inflammatory activity of compound **88** was better than that of compound **86** indicating the important role of hydroxy group on C-7. Moreover, compounds containing methylene alcohol on C-3 (**86**, **88**, and **90**) showed more potent anti-inflammatory activity compared with the derivatives with carboxylic acid functional groups (**87** and **89**). Four Eremophilane sesquiterpenoids (**91**–**94**) were isolated from deep-marine-sediment-derived fungus *Aspergillus sp.* SCSIOW2, and all showed inhibitory activity of NO production in a dose-dependent manner [49]. Additionally, five sesquiterpenoids (**95**–**99**) were isolated from the mangrove endophytic fungus *Aspergillus sp.* GXNU-MA1 by Zhou et al., which exhibited moderate inhibitory activities against NO production, with IC_50_ values ranging from 16.15 to 27.08 µM [50]. Niu et al. isolated six phenolic bisabolane (**100**–**105**) and two cuparene sesquiterpenoids (**106**–**107**) from *Aspergillus sydowii* MCCC3A00324 derived from deep sea sediments [51]. Compounds **100**, **101**, and **103**–**105** showed anti-inflammatory activity against NO secretion in LPS-activated BV-2 microglia cells, with the inhibition rates of more than 45% at 10 µM, while those of compounds **102**, **106**, and **107** were 32.8%, 32.6% and 45.4%, respectively. Furthermore, compound **101** exerted an anti-inflammatory effect by inhibiting NF-κB activation pathway in a dose-dependent manner. Tan et al. isolated a new nitrobenzoyl sesquiterpenoid (**108**) from *Aspergillus ochraceus*, which could suppress the RANKL-induced osteoclats formation and bone resorption by targeting NF-κB [52]. Additionally, compound **108** attenuated inflammatory bone loss in vivo.

### 3.4. Enzymatic Inhibitory Activity

**Figure 8:**
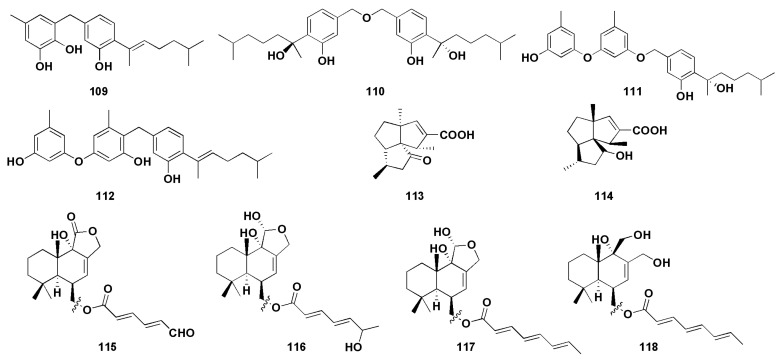
Chemical structures of enzymatic inhibitory compounds (**109**–**118**).

Enzyme inhibitors are of value in treating many diseases in clinical use, and have become a very attractive target for drug development and discovery. In recent years, the prominence of various enzyme inhibitors has been discussed extensively by many researchers in comprehensive systematic reviews [72]. In this section, the inhibitory activities of sesquiterpenoids (Figure 8) from marine *Aspergillus* sp. against three enzymes (α-glucosidase, cholinesterase, and neuraminidase) are briefly reviewed.

α-Glucosidase is a membrane-bound enzyme present in the small intestinal epithelium [73], whose role is to promote the absorption of glucose in the small intestine by catalyzing the hydrolysis of oligosaccharides into absorbable glucose. α-Glucosidase inhibitors are the most widely used drugs in the clinical treatment of diabetes in China. By inhibiting the activity of α-glucosidase, the formation and absorption of glucose can be reduced to achieve the goal of lowering blood glucose. At the same time, it can also reduce the stimulation of blood glucose on the pancreas, effectively preventing and relieving diabetic complications [74]. 7-Deoxy-7,14-didehydrosydonol (**79**) was found from the mangrove endophytic fungus *Aspergillus versicolor* and possessed a significant inhibitory effect on α-glucosidase, with an IC_50_ value of 7.5 μM (acarbose as 350 μM), and the terminal ethylene group at C-7 may play a key role in α-glucosidase inhibition activity [45]. Wu et al. [53] isolated four phenolic bisabolane sesquiterpenoids (**109**–**112**) from the mangrove endophytic fungus *Aspergillus flavus* QQSG-3. The inhibitory activity studies of α-glucosidase showed that the compounds (**109**–**112**) had strong inhibitory effects, with IC_50_ values of 4.5, 3.1, 1.5, and 2.3 µM, respectively (all lower than the positive control drug acarbose).

Alzheimer’s Disease (AD) is a degenerative disease with unknown causes, mainly involving cerebral cortical neurons, which is the major cause of dementia [75]. The currently accepted pathogenesis is the cholinergic deficiency hypothesis [76]. Cholinesterase inhibitors (ChEI) are a class of drugs that can bind to cholinesterase (ChE) and inhibit ChE activity; they are also approved as first-line drugs for the treatment of mild-to-moderate AD [77]. Feng et al. firstly isolated the potential reversible cholinesterase inhibitor cyclopentapentalane sesquiterpenoid subergorgic (**113**) and its analogues 2-deoxy-2β-hydroxysubergorgic (114) from the soft-coral-derived fungus *Aspergillus* sp. EGF15-0-3 [54].

Neuraminidase (NA) is the most critical enzyme for influenza virus replication and diffusion in host cells and has become an important target for anti-influenza virus drug design [78]. Li et al. [55] isolated four drimane sesquiterpenoids (**115**–**118**) from the ascidian endophytic fungus *Aspergillus ustus* TK-5, which showed significant inhibitory activity against neuraminidase, with IC_50_ values of 31.8, 37.3, 28.4, and 36.8 µM, respectively. Further results showed that the degree of unsaturation of 11-OH and C-6 linked side chains, which can improve their neuraminidase inhibitory activity.

### 3.5. Other Activities

**Figure 9:**
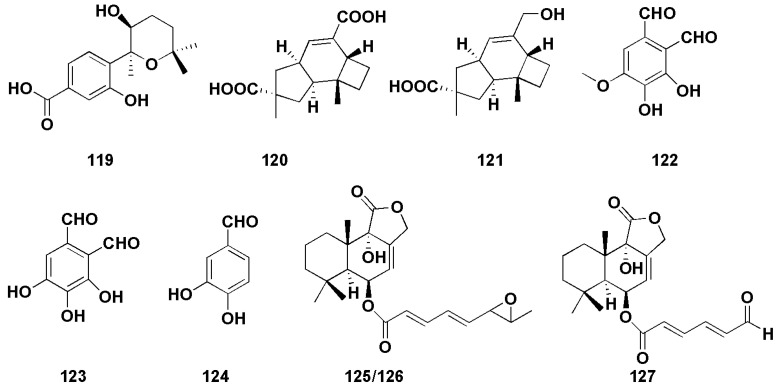
Chemical structures of other biological compounds (**119**–**127**).

Hu et al. isolated an aromatic bisabolane sesquiterpenoid (7S,8S)-8-hydroxysydowic acid (**119,** Figure 9)) from the marine red algae endophytic fungus *Aspergillus sydowii* EN-434, which exhibited DPPH free radical scavenging activity, with an IC_50_ value of 113.5 µM [79]. An et al. found two sesquiterpenoids (**120**–**121,** Figure 9) with weak DPPH radical scavenging activity, with IC_50_ values of 1.8 mM and 0.6 mM, respectively (V_C_ as 0.04 mM) [80]. Zhong et al. isolated three sesquiterpenoids (**122**–**124,** Figure 9) from the marine-offshore-mud-derived fungus *Aspergillus pseudoglaucus* [81]. Among them, compounds **122** and **123** showed strong DPPH radical scavenging activity, with IC_50_ values of 2.42 and 1.86 μg/mL (V_C_ was 3.25 μg/mL), respectively, while compound **124** exhibited moderate antioxidant activity (IC_50_ was 10.89 μg/mL).

Two bisabolane-type sesquiterpenoids (**4**–**5**) were derived from sponge-derived fungus *Aspergillus* sp., among which compound **4** completely inhibited larval settlement at 25.0 μg/mL, while compound **5** displayed an obvious toxic effect on larvae at the same concentration [14]. Compound **7** also showed weak anti-H_3_N_2_ activity, with IC_50_ values of 57.4 μM [15]. (−)-(7S)-10-hydroxysydonic acid (**28**) was found to have a mild DPPH radical scavenging activity, with an IC_50_ value of 72.1 μM [21]. Nitrobenzoyl sesquiterpenoids (**49**) also showed moderate antiviral activities against H_3_N_2_ and EV71, with IC_50_ values of 17.0 and 9.4 μM, respectively [30]. Liu et al. [82] isolated three drimane sesquiterpenoids (**125**–**127**, Figure 9) from the marine-green-alga-derived fungus *Aspergillus ustus*. In the brine shrimp (*Artemia salina*) toxicity assay, there was more than 75% lethality at the concentration of 100 μg/mL, and the LC_50_ values were 41.8, 62.2 and 48.9 μg/mL, respectively.

## 4. Chemical Synthesis and Biosynthesis of Sesquiterpenoids from Marine *Aspergillus* sp.

### 4.1. Chemically Induced Synthesis

**Figure 10:**
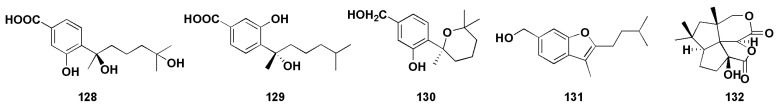
Structures of sesquiterpenoids obtained from chemical synthesis and biosynthesis from the *Aspergillus* sp. (**128**–**132**).

*Aspergillus* sp. is the important source for the discovery of natural active products with novel and diverse structures. However, in recent years, the continual study of secondary metabolites of marine fungi has led to a high frequency of repeated discovery of known compounds [83]. This encourages us to develop new strategies to obtain new natural products. Studies have found that a large number of secondary metabolite biosynthesis gene clusters exist in the genome of *Aspergillus* fungi. Furthermore, the genome can be segmented into active and silent clusters, while the silent clusters are inactive under normal environmental conditions [84,85,86]. In order to obtain more active metabolites, researchers have applied a variety of methods to activate silenced biological genetic gene clusters, such as transcription factor regulation, targeted genome mining, heterologous expression of gene clusters, and chemical epigenetic regulation [87,88,89]. Because of its simplicity and effectiveness, chemical epigenetic regulation has been widely used in marine fungi to activate silenced gene clusters, which could lead to the production of new secondary metabolites or known components with a higher concentration. Wang et al. [90] cultivated the gorgonian-derived fungus *Aspergillus* sp. SC-20090066 with a DNA methyltransferase inhibitor 5-azacytidae (5-AZA) in the culture medium and led to the isolation of six new bisabolane-type sesquiterpenoids (Figure 10). Among them, compounds (**128**–**130**) exhibited broad spectrum activities against *S. aureus*, *Bacillus cereus*, *Rhizophila*, *Pseudomonas putida*, and *Pseudomonas aeruginosa*, with MICs of less than 25 μM. In particular, compound **130** exhibited significant antibacterial activity against *S. aureus*, with MIC value of 3.13 μM, which was close to the positive control ciprofloxacin (MIC value was 2.5 μM). In order to trigger the chemical diversity of marine-derived fungus *Aspergillus versicolor* XS-2009006, epigenetic agents (histone deacetylase inhibitor SAHA and DNA methyltransferase inhibitor 5-AZA) were added to the culture medium by Wu et al. [91] Interestingly, the secondary metabolites was significantly increased and a new bisabolane sesquiterpene aspergillusene E (**131, Figure 10**) was isolated, which showed anti-larval attachment activity against bryozoan *B. neritina*, with the EC_50_ and (lethal concentration 50%) LC_50_ values of 6.25 μg/mL and 25 μg/mL, respectively. In addition, compound **131** showed certain antibacterial activities against *Staphylococcus epidermidis* and *S. aureus*, with MIC values ranging from 8 to 16 μM. By adding DNA methyltransferase inhibitors to the medium of *Aspergillus sydowii*, the composition of secondary metabolites was further changed and new bisabolane sesquiterpenoids (**86**–**87**) were isolated [48]. In addition, Wang et al. [49] applied chemical epigenetic manipulation to *Aspergillus* sp. SCSIOW2 and obtained four eremophilane sesquiterpenes with anti-inflammatory activity (**91**–**94**).

### 4.2. Biosynthetic Pathways

The skeleton structures of sesquiterpenoids were derived from farnesyl diphosphate (FPP) and underwent a series of reaction steps, including intramolecular rearrangement, cyclysis, and other biosynthetic transformations, leading to their structural diversity [92]. Ingavat et al. [93] studied the proposed biosynthesis of sesquiterpene compound **132** in *Aspergillus aculeatus*, which starts from a double-bond migration (C1/C2 to C2/C3) of silphineneene intermediate 2, and then the double bond of C2/C3 undergoes oxidative cleavage to generate intermediate 3, which, in turn, undergoes a series of oxidation and lactonizations to finally give **132** (Figure 10).

Wang et al. [46] proposed a biogenetic pathway for the synthesis of aspertetranones A-D (**80**–**83**). Common drimane-type merosesquiterpene were obtained by cyclization of farnesylated pyrone, followed by oxidation and retro-aldo/aldo rearrangement to produce the unique terpenoid part of aspertetranones. After nucleophilic attack and dehydration, the leaborate preaspertetranone was obtained. Illudalanes derive biosynthetically from a humulene precursor after cyclization, producing a protoilludanes, which is eventually rearranged to form the irudane derivative [94]. According to this report, Orfali et al. speculated a biosynthetic pathway of asperorlactone (**41**), in which illudol was a key intermediate. The iluane-type sesquiterpene asperorlactone can be synthesized by dehydration, oxidation, and four-membered ring opening [27].

## 5. Potency of Sesquiterpenoids from Marine *Aspergillus* sp.

Secondary metabolites of microorganisms in the marine environment, mainly derived from marine fungi, are a great source for new drug screening. Currently, the marine drug library includes 15 approved drugs (primarily for cancer treatment), 7 phase I compounds, 12 phase II compounds, and 5 compounds in phase III clinical trials, the latter including a recently recommended drug for symptomatic treatment of COVID-19 (Plitidepsin) [95,96]. Compound **13** displayed significant inhibitory activity against *E. coli* (MIC 1.0 μg/mL), and its antibacterial effect was more potent than that of the positive control chloramphenicol (MIC 2.0 μg/mL), which was expected to be a lead compound for antibiotics [17]. The sesquiterpene compound (**79**) isolated from *Aspergillus versicolor* exhibited better inhibitory effect on α-glucosidase than acarbose, while its anti-inflammatory effect was also stronger than that of indomethacin [45]. Compound **88** derived from marine sediments, showed a significant anti-inflammatory effect and hypoglycemic effect. In addition, compound **88** could also inhibit fat accumulation in adipocytes [48]. These results indicated compound **79** and **88** has the potential to be a lead compound targeting the vicious diabetes-inflammation cycle. Feng et al. found that sesquiterpene compound **113**, the reversible cholinesterase inhibitor, is a promising new drug candidate for the treatment of Alzheimer’s Disease and a preclinical trial is already under way [54].

## 6. Conclusions and Perspective

In this paper, the biosources, bioactivities, structural types, biosynthetic, and pharmacogenic potential of sesquiterpenoids found from marine fungi *Aspergillus* sp. were reviewed. A total of 268 sesquiterpenes were isolated, including 131 bioactive sesquiterpenes, most of which were bisabolanes, followed by drimanes and nitrobenzoyl, etc. Most *Aspergillus* species derived from sponges, marine sediments, algae, mangroves, corals, etc. The main *Aspergillus* species involved are as follows: *Aspergillus fumigatus*, *Aspergillus versicolor*, *Aspergillus flavus*, *Aspergillus ustus*, *Aspergillus sydowii*, and so on. These sesquiterpenes exhibited excellent pharmacological activities such as antibacterial, antitumor, anti-inflammatory, and enzyme inhibitory activities. Additionally, the biosynthesis and total synthesis of sesquiterpenes derived from marine *Aspergillus* sp. have also promoted the in-depth understanding of these sesquiterpenes. Because of the chemical and biological activity of these sesquiterpenoids, it is worthwhile to find promising lead compounds for the development of marine drugs in further studies from marine fungi.

## Figures and Tables

**Table 1:** List of sesquiterpenoids isolated from marine fungi *Aspergillus* sp. with potential biological activity.

Compound Name/Chemical Class	Marine Source	Type of Strains	Activity (MIC)	Reference
Compounds **1**,**3** and **5**	Marine-sponge-derived fungus *Aspergillus*	not reported	not reported	[14] 2012
Compound **2**		*S. albus*, *M. tetragenus*	1.25–5 µM	
Compound **4**		*S. albus*, *B. subtilis*	2.5–5 µM	
Compound **6**	Marine-sponge-derived fungus *Aspergillus sydowii* ZSDS1-F6	*A. hydrophila* and *K. pneumonia*	4.3 and 21.4 µM	[15] 2014
Compound **7**		*K. pneumonia*	10.7 µM	
Compound **8**		*E. faecalis*	18.8 µM	
Flavilane A(**9**)	Fresh-seawater-derived fungus *Aspergillus flavipes* 297	Pathogenic bacteria	2–64 µM	[16] 2021
Compound **10**		Pathogenic bacteria and Pathogenic fungus *V. mari*		
Compounds **11**,**12**	Deep sea sediment fungus *Aspergillus versicolor* SD-330	*A. hydrophilia*, *E. coli*, *E. tarda*, and *V. harveyi*	2–8 µM	[17] 2021
Compound **13**		*E. coli*	1 µM	
Compound **14**	Seawater-derived fungus *Aspergillus sydowii* SW9	*E. coli* and *S. pneumonise*	2–4 µM	[18] 2019
Compounds **15**,**16**	Deep sea sediment fungus *Aspergillus versicolor* SD-330	*E. coli*, *E. trada*, *V. harveyi*, and *V. parahaemolyticus*	8 µM	[19] 2019
Compound **17**		*E. coli*, *Aeromonas hydrophilia*, *E. tarda*, *V. anguillarum*, and *V. harveyi*	1–4 µM	
Compound **18**		not reported	not reported	
Compounds **19**–**21**	Marine-gorgonian-derived fungus *Aspergillus*	*S. aureus*	Inhibition zones were 5–11 mm at 100µg/mL	[20] 2010
Compounds **22**–**28**	Mangrove endophytic fungus *Aspergillus* xy02	*S. aureus*	31.5–41.9 µM	[21] 2018
Asperchondols A, B(**29**, **30**)	Marine-sponge-derived fungus *Aspergillus*	*S. aureus*	25–50 µM	[22] 2016
Albican-11,14-diol (**31**)	Marine-alga-derived fungus *Aspergillus versicolor*	*E. coli* and *S. aureus*	Inhibition zones were 7–10.3 mm at 30 μg/disk	[23] 2012
Compounds **32**–**35**	Marine-alga-derived fungus *Aspergillus* RR-YLW-12	*V. harveyi*, *V. splendidus, V. parahaemolytics*, and *V. anguillarum*	not reported	[24] 2021
Compounds **36**–**38**	Marine-coral-derived fungus *Aspergillus versicolor* ZJ-2008015	*S. aureus* and *S. albus*	2.6–6.4 µM	[25] 2012
Compounds **39**,**40**	Marine-sponge-derived fungus *Aspergillus insuetus* OY-207	*N. crassa*	140–242 µM	[26] 2011
Compound Name/Chemical Class	Marine Source	Cell lines	Activity (IC_50_/EC_50_/ED_50_/inhibition rate)	Reference
Asperorlactone (**41**)	Marine sediment fungus *Aspergillus oryzae*	A549, HepG2, and MCF-7	<100 µM	[27] 2021
Echinolactone D (**42**)			not reported	
Asperienes A-D (**43**–**46**)	Marine fungus *Aspergillus flavus* CF13–11	A549, HeLa, MGC-803, and MCF-7	1.4–8.3 µM	[28] 2019
Compounds **47**,**48**	Marine sediment fungus *Aspergillus flocculosus*	Neuro-2a and 22Rv1	3–31.5 µM	[29] 2019
Compounds **49**,**50**	Marine fungus *Aspergillus ochraceus* Jcma1F17	H1975, U937, K562, BGC-823, MOLT-4, MCF-7 A549, HeLa, HL60, and Huh-7	1.95–6.35 µM	[30] 2014
Insulicolide A (**51**)	Marine-sponge-derived fungus *Aspergillus insulicola* MD10-2	H-460	6.9 µM	[31] 2016
Compounds **52**, **53**	Marine fungus *Aspergillus ochraceus* Jcma1F17	786-O, ACHN, and OS-RC-2	2.3–11 µM	[32] 2018
Compound **54**			0.89–1.5 µM	
Compounds **57**, **58**	Marine-sponge-derived fungus *Aspergillus insulicola*	AsPC-1 and PANC-1	2.3–4.6 µM	[33] 2022
Compounds **59**, **61**	Marine-sponge-derived fungus *Aspergillus ustus*	L5178Y	0.6–5.3 µM	[34] 2009
Compound **60**		L5178Y, PC12, and HeLa	0.6–7.2 µM	
Compound **62**	Mangrove endophytic fungus *Aspergillus ustus*	P388	8.7 µM	[35] 2011
Compounds **63**, **65**	Marine-sponge-derived fungus *Aspergillus*	HePG-2 and Caski	2.91–12.4 µM	[36] 2012
Compound **64**			not reported	
β-D-glucopyranosylaspergillusene A (**66**)	Marine-sponge-derived fungus *Aspergillus sydowii* J05B7F-4	HePG-2, HCT116, and KB	50–70 µM	[37] 2017
Compound **67**	Mangrove endophytic fungus *Aspergillus terreus* GX3-3B	MCF-7, HL-60	3.43–4.49 µM	[38] 2013
Compound **68**		MCF-7	2.79 µM	
Compound **70**		HL-60	0.6 µM	
Aspergiketone (**71**)	Coastal saline soil fungus *Aspergillus fumigatus*	HL-60 and A549	12.4–22.1 µM	[39] 2016
Oxalicine B (**72**)	Sea-urchin-derived fungus *Aspergillus fumigatus*	P388	55.9 µM	[40] 2012
Compound **73**	Marine fungus *Aspergillus ustus* 094102	HL-60 and A549	20.6–30 µM	[41] 2009
Compounds **74**, **75**			9–10.5 µM	
Compound **76**	Marine-sponge-derived fungus *Aspergillus ustus*	L5178Y	1.9 µM	[42] 2008
Compound **77**	Marine sediment fungus *Aspergillus fumigatus* YK-7	U937	84.9 µM	[43] 2015
Asperflavinoid A (**78**)	Marine fungus *Aspergillus flavipes* 297	HepG2 and MKN-45	26.8–38.5 µM	[44] 2021
Compound Name/Chemical Class	Marine Source	Target Enzyme	Activity (IC_50_/inhibitory rate)	Reference
7-Deoxy-7,14-didehydrosydonol (**79**)	Mangrove endophytic fungus *Aspergillus versicolor* SYSU-SKS025	inhibit NO production in RAW 264.7 macrophages	12.5 µM	[45] 2018
Compounds **80**–**82**	Marine-algal-derived fungus *Aspergillus* ZL0-1B14	inhibit LPS-stimulated RAW264.7 macrophages	not reported	[46] 2015
Compound **83**		inhibit LPS-stimulated RAW264.7 macrophages and exhibited an inhibitory effect against IL-6 production	69% at 40 µM	
Compound **84**,**85**	Marine fungus *Aspergillus terreus*	inhibitory activity of NO production	37.3% and 47.7% at 40 μM	[47] 2018
Compound **86**,**87**,**89**	Marine sediment fungus *Aspergillus sydowii*	not reported	not reported	[48] 2013
Compounds **88**,**90**		inhibition against fMLP/CB-induced superoxide anion generation by human neutrophils and inhibitory activity against the release of elastase induced by fMLP/CB	5.23–16.39 µM	
Compound **91**–**94**	Marine sediment fungus *Aspergillus* SCSIOW2	inhibitory activity of NO production	not reported	[49] 2016
Compounds **95**–**99**	Mangrove endophytic fungus *Aspergillus* GXNU-MA1	inhibitory activity of NO production	16.15–27.08 µM	[50] 2022
Compounds **100**,**102**–**107**	Marine sediment fungus *Aspergillus sydowii* MCCC3A00324	against NO secretion in LPS-activited BV-2 microglia cells	32.6%-45.4% at 10 µM	[51] 2020
Compound **101**		against NO secretion in LPS-activited BV-2 microglia cells and anti-inflammatory effect	45% at 10 µM	
		inhibiting NF-κB activation pathway	not reported	
Compound **108**	Marine fungus *Aspergillus ochraceus*	suppressed the RANKL-induced osteoclats formation and bone resorption by targeting NF-κB	not reported	[52] 2020
Compound Name/Chemical Class	Marine Source	Target Enzyme	Activity/(IC_50_)	Reference
7-Deoxy-7,14-didehydrosydonol (**79**)	Mangrove endophytic fungus *Aspergillus versicolor* SYSU-SKS025	inhibitory effect on α-glucosidase	7.5 µM	[45] 2018
Compounds **109**–**112**	Mangrove endophytic fungus *Aspergillus flavus* QQSG-3	inhibitory effect on α-glucosidase	1.5–4.5 µM	[53] 2018
Compound **113**	Marine-coral-derived fungus *Aspergillus* EGF15-0-3	inhibit ChE	not reported	[54] 2020
2-deoxy-2β-hydroxysubergorgic (**114**)				
Compounds **115**–**118**	Marine-ascidian-derived fungus *Aspergillus ustus* TK-5	inhibitory activity against neuraminidase	28.4–37.3 µM	[55] 2018
